# The rationale for Health and Demographic Surveillance System (HDSS) in urban populations in Ethiopia

**DOI:** 10.4314/ejhs.v34i2.1S

**Published:** 2024-12

**Authors:** Yemane Berhane, Semira Abdelmenan, Alemayehu Work

**Affiliations:** 1 Department of Epidemiology and Biostatistics, Addis Continental Institute of Public Health, Addis Ababa, Ethiopia

Accurate population-level information is critical for planning, implementing, and evaluating social and economic interventions. Adequate information on population size, age, and sex distribution helps design social interventions. Short- and long-term planning relies on accurate population-level information on trends and projections.

The lack of a complete and dynamic civil registration system in low-middle-income countries (LMICs) triggered alternative approaches to gathering population-level data. One such approach was to use a Demographic Surveillance System (DSS) or Health and Demographic Surveillance System (HDSS) that regularly updates population details by registering births, deaths, marriage, and migration by visiting every household in a designated geographic location.

The establishment of HDSS globally started in the late 1950s, notably after the establishment of the MATLAB population lab in Bangladesh. The rationale for establishing HDSS was to provide evidence of the effectiveness of primary healthcare intervention, including oral rehydration therapy, bed nets for malaria prevention, and childhood vaccination (1). Since then, many more HDSS/DSS sites have been established in Africa and South-East Asia (2). The establishment of the INDEPHTH network in the late 1990s helped HDSSs to standardize methodologies and data structures. In Ethiopia, Addis Ababa University established the first HDSS in Butajira in 1986, in central Ethiopia (3). Currently, many of the senior universities in Ethiopia have HDSS sites. Most HDSS/DSS sites in Ethiopia and sub-Saharan Africa are typically located in rural populations. The Harar Urban HDSS, established in 2011, is Ethiopia's first urban Health and Demographic Surveillance System site (4). The need for urban sites cannot be overemphasized with the increasing urban population size and fast-changing population dynamics in LMICs. Urban HDSSs offer several advantages, particularly in the context of rapidly urbanizing populations. By capturing data from urban populations, which are often underrepresented in most health and demographic research, these systems help in understanding urban health dynamics and planning appropriate interventions.

The HDSS is an excellent platform for training epidemiologists and public health practitioners to better understand epidemiological indicators, population dynamics trends, and the effectiveness of public health interventions. In the long run, HDSS data can be used to determine the cause of death and mortality patterns in the population (5,6). Changes in cause-specific mortality rates can provide evidence of the effectiveness of preventive interventions such as immunization and early detection (7). HDSS can depict local migration patterns useful for organizing routine services during epidemics/pandemics (8).

Having an HDSS in LMICs has many advantages. First, it provides population information necessary for predicting population dynamics. Second, the HDSS can serve as a sampling frame for epidemiological studies. Third, it can be a platform for interventional studies (trials). The HDSS also has some notable limitations: although population-based, it may not represent the national or sub-nation levels. HDSS can trigger community fatigue, leading to information inaccuracy due to response biases.

The Addis Health and Demographic Surveillance System (Addis-HDSS) was established by the Addis Continental Institute of Public Health in Addis Ababa, Ethiopia. Addis Ababa is Ethiopia's capital, with an estimated population of over four million. However, the population size and composition are calculated based on a 2007 census and cannot accurately reflect the population dynamics that have occurred since. Thus, having an HDSS in such a large and dynamic city is essential to generating public health indices (5).

This special issue of the Ethiopian Journal of Health Sciences publishes the context and methods as well as some valuable population-based data on Addis Ababa city, including the household sanitation and crowding Status, an alternative single-item socioeconomic tool for categorizing urban population into economic strata, household healthcare facility preferences when a family member is sick, self-rated health status of the adult population, population nutritional status, socioeconomic determinants of contraceptive use among married women, prevalence of self-reported chronic non-communicable diseases among adults, and prevalence of reported mental illness. The supplement's information can be considered a bird's eye view of the potential of this HDSS as more evidence is expected to be generated from subsequent rounds. The HDSS has already hosted several studies on pressing public health issues, such as infant feeding practices and adolescent health. This evidence and those to be generated in the future would help improve the quality of public health interventions in the city. It can benchmark interventions in similar urban areas in Ethiopia and other LMICs.

## References

[1] Herbst K, Juvekar S, Jasseh M Health and demographic surveillance systems in low- and middle-income countries: history, state of the art and future prospects. Global Health Action.

[2] Network I (2002). Population and health in developing countries: volume 1; population, health, and survival at INDEPTH sites.

[3] Berhane Y, Wall S, Kebede D, Emmelin A, Enquselassie F, Byass P (2000). Establishing an epidemiological field laboratory in rural areas-potentials for public health research and interventions The Butajira Rural Health Programme 1987-1999. Ethiopian Journal of Health Development.

[4] Harar CHAMPS Catchment Area | Hararghe Health Research Partnership https://hararghe.org/node/9.

[5] Byass P, Berhane Y, Emmelin A (2002). The role of demographic surveillance systems (DSS) in assessing the health of communities: an example from rural Ethiopia. Public Health.

[6] Kabudula CW, Houle B, Ohene-Kwofie D (2021). Mortality transition over a quarter century in rural South Africa: findings from population surveillance in Agincourt 1993-2018. Global Health Action.

[7] Ashenafi W, Eshetu F, Assefa N (2017). Trend and causes of adult mortality in Kersa health and demographic surveillance system (Kersa HDSS), eastern Ethiopia: verbal autopsy method. Population Health Metrics.

[8] Byass P, Berhane Y, Emmelin A, Wall S (2003). Patterns of local migration and their consequences in a rural Ethiopian population. Scandinavian Journal of Public Health.

